# Novel Reassortant Highly Pathogenic Avian Influenza (H5N8) Virus in Zoos, India

**DOI:** 10.3201/eid2304.161886

**Published:** 2017-04

**Authors:** Shanmugasundaram Nagarajan, Manoj Kumar, Harshad V. Murugkar, Sushil Tripathi, Shweta Shukla, Sonam Agarwal, Garima Dubey, Raunaq Singh Nagi, Vijendra Pal Singh, Chakradhar Tosh

**Affiliations:** Indian Council of Agricultural Research– National Institute of High Security Animal Diseases, Bhopal, India

**Keywords:** highly pathogenic avian influenza virus, HPAI, waterfowl, subtype H5N8, phylogenetic analysis, clade 2.3.4.4, India, influenza, viruses, zoos, zoonoses

## Abstract

Highly pathogenic avian influenza (H5N8) viruses were detected in waterfowl at 2 zoos in India in October 2016. Both viruses were different 7:1 reassortants of H5N8 viruses isolated in May 2016 from wild birds in the Russian Federation and China, suggesting virus spread during southward winter migration of birds.

Since 1996, the hemagglutinin (HA) gene of subtype H5N1 highly pathogenic avian influenza (HPAI) viruses has evolved into multiple phylogenetic clades (*1*). During 2010, subtype H5N8 virus, bearing an H5N1 backbone and polymerase basic (PB) protein 1 (PB1), nucleoprotein (NP), and neuraminidase (NA) genes from non-H5N1 virus, emerged in China (*2*). In January 2014, a novel reassortant HPAI (H5N8) virus was detected in poultry and wild birds in South Korea (*3*) and subsequently spread to other counties in Asia and Europe before reaching North America by the end of 2014 (*4*). Because the H5N8-associated outbreaks coincided with bird migration routes, movement of wild waterfowl was suspected in intercontinental spread (*5*). Therefore, understanding the source and spread of the virus is a critical requirement for guidance of control measures. We report analysis of the genome of HPAI (H5N8) viruses isolated from waterfowl (domestic duck [*Anas platyrhynchos domesticus*] and painted stork [*Mycteria leucocephala*]) at 2 zoos in India in October 2016.

Twenty avian influenza viruses were isolated from 83 samples from National Zoological Park, Delhi, and Gandhi Zoological Park, Gwalior, Madhya Pradesh, India, in October 2016. The viruses were subtyped as H5N8 by using reverse transcription PCR and real-time RT-PCR (Technical Appendix 1). One representative isolate each from Delhi (A/duck/India/10CA01/2016) and Madhya Pradesh (A/painted stork/India/10CA03/2016) were processed for pathogenic and molecular characterization. A detailed description of the methods for the intravenous pathogenicity index test and genetic analysis used are provided in Technical Appendix 1. Nucleotide sequences were deposited in the GISAID EpiFlu database (http://www.gisaid.org) under accession nos. EP1858833–EP1858848.

Both isolates were highly pathogenic based on amino acid sequence at the HA cleavage region (PLREKRRKR/GLF), which was corroborated by using an intravenous pathogenicity index test of 3.00 (Delhi isolate) and 2.96 (Madhya Pradesh isolate). Amino acid markers in the neuraminidase protein and matrix protein 2 indicated sensitivity to neuraminidase inhibitors and amantadines. Markers for mammalian virulence and poultry adaptation, such as E627K and D701N in PB2 and amino acid deletion in nonstructural protein (NS) 1 (position 80–84), were absent in the H5N8 viruses. However, 42S and 13P mutations in NS and PB1 genes (*6*) associated with increased virulence of the virus to mice were present. The PB1-F2 protein was truncated because of nucleotide mutation C35A, leading to premature termination after 11 aa. 

Except the polymerase acidic (PA) and NP genes, all other gene segments of both isolates shared high nucleotide identity, ranging from 99.2% to 99.5%. The nucleotide identity of the PA and NP gene was 95.8% and 94.8%, respectively, suggesting involvement of 2 gene pools of H5N8 virus in the waterfowl outbreaks at Delhi and Madhya Pradesh. 

In the HA gene phylogeny, the Indian isolates clustered with H5N8 viruses from other countries in Asia and Europe within group B (intercontinental group B) (Technical Appendix 1 Figures 1–8). A similar grouping pattern was observed in the neuraminidase and nonstructural (NS) gene phylogenies. Further, within intercontinental group B, the isolates shared >99% nucleotide sequence identity with H5N8 viruses isolated in Uvs-Nuur Lake (located at the Mongolia–Russia border) and Qinghai Lake, China, in May 2016 (Technical Appendix 1 Table 2). However, PB1, PB2, and matrix protein genes grouped with low pathogenic avian influenza (LPAI) viruses isolated in Eurasia and H5N8 viruses isolated in Qinghai Lake, Uvs-Nuur Lake, and Tyva Republic (Russian Federation). 

In the PA phylogeny, although the Delhi virus grouped with LPAI viruses isolated in Mongolia and Vietnam and viruses isolated in Qinghai Lake, Uvs-Nuur Lake, and Tyva Republic, the Madhya Pradesh virus shared close relationship with LPAI viruses from Eurasia. In the NP gene phylogeny, the Delhi virus shared close relationship with the Eurasia group of LPAI, whereas the Madhya Pradesh virus and H5N8 viruses from Qinghai Lake, Uvs-Nuur Lake, and Tyva Republic are closely related to the Eurasia 2 LPAI viruses. These results suggest that both isolates are 7:1 reassortant of the Tyva Republic and Uvs-Nuur Lake H5N8 viruses reported previously (*7*) with different gene constellations. A median-joining network analysis indicated that, even though the contemporary H5N8 viruses isolated from wild birds in Qinghai Lake, Uvs-Nuur Lake, and Tyva Republic are not the direct ancestors, closely related precursor gene pools are source of the H5N8 viruses that caused outbreaks in waterfowls at the 2 zoos in India (Technical Appendix 1 Figure 9).

The outbreak in waterfowls at both zoos coincided with winter migration of birds to India (September–March). The Uvs-Nuur Lake is an important habitat for 46 resident waterfowl species and 215 different species of birds migrating southward from Siberia (*8*). Therefore, different waves of migration of the wild birds might be the source of introduction of the H5N8 virus at the 2 zoos in India, as suggested by the observed spread of H5N1 clade 2.2 and 2.3.2.1c viruses (*9*,*10*).

Technical Appendix 1 Materials and methods for clinical sample collection, virus isolation, and laboratory confirmation for the analysis of novel reassortant highly pathogenic avian influenza virus subtype H5N8 implicated in outbreaks among waterfowl at 2 zoos in India. Summary of avian influenza H5N8–positive samples. Maximum-likelihood phylogenetic trees for all 8 genes. Comparison of nucleotide sequence homology of H5N8 highly pathogenic avian influenza virus isolates in India compared with other available sequences in the database. Median-joining network analysis based on the hemagglutinin gene.

Technical Appendix 2 Acknowledgments of researchers for the avian influenza virus nucleotide sequences submitted to the Global Initiative on Sharing All Influenza Data EpiFlu Database.

## References

[1] WHO/OIE/FAO H5N1 Evolution Working Group. Continued evolution of highly pathogenic avian influenza A(H5N1): updated nomenclature. Influenza Other Respi Viruses. 2012;6:1–5 . 10.1111/j.1750-2659.2011.00298.x 22035148 PMC5074649

[2] Zhao K, Gu M, Zhong L, Duan Z, Zhang Y, Zhu Y, et al. Characterization of three H5N5 and one H5N8 highly pathogenic avian influenza viruses in China. Vet Microbiol. 2013;163:351–7. 10.1016/j.vetmic.2012.12.025 23375651

[3] Lee YJ, Kang HM, Lee EK, Song BM, Jeong J, Kwon YK, et al. Novel reassortant influenza A(H5N8) viruses, South Korea, 2014. Emerg Infect Dis. 2014;20:1087–9. 10.3201/eid2006.140233 24856098 PMC4036756

[4] World Organization for Animal Health. Summary of immediate notifications and follow-ups—2014: highly path. avian influenza [cited 2016 Dec 3]. http://www.oie.int/wahis_2/public/wahid.php/Diseaseinformation/Immsummary

[5] Lee DH, Torchetti MK, Winker K, Ip HS, Song CS, Swayne DE. Intercontinental spread of Asian-origin H5N8 to North America through Beringia by migratory birds. J Virol. 2015;89:6521–4. 10.1128/JVI.00728-15 25855748 PMC4474297

[6] Gabriel G, Dauber B, Wolff T, Planz O, Klenk HD, Stech J. The viral polymerase mediates adaptation of an avian influenza virus to a mammalian host. Proc Natl Acad Sci U S A. 2005;102:18590–5. 10.1073/pnas.0507415102 16339318 PMC1317936

[7] Lee D-H, Sharshov K, Swayne DE, Kurskaya O, Sobolev I, Kabilov M, et al. Novel reassortant clade 2.3.4.4 highly pathogenic avian influenza A(H5N8) virus in wild aquatic birds, Russia, 2016. Emerg Infect Dis. 2017 Feb 15 [Epub ahead of print]. 10.3201/eid2302.161252 PMC5324796 27875109

[8] Florin BM. Uvs Nuur, Lake. In: Robert W. H. ed. Biomes and Ecosystems. Vol. 4. Amenia (NY): Salem Press; 2013. p. 1260–1.

[9] Chen H, Smith GJ, Zhang SY, Qin K, Wang J, Li KS, et al. Avian flu: H5N1 virus outbreak in migratory waterfowl. Nature. 2005;436:191–2. 10.1038/nature03974 16007072

[10] United Nations Food and Agriculture Organization. H5N8 highly pathogenic avian influenza (HPAI) of clade 2.3.4.4 detected through surveillance of wild migratory birds in the Tyva Republic, the Russian Federation—potential for international spread [cited 2016 Dec 3]. http://www.fao.org/3/a-i6113e.pdf

