# Supplementary material for: Novel Reassortant Highly Pathogenic Avian Influenza (H5N8) Virus in Zoos, India
Source: Emerg Infect Dis. 2017 Apr;23(4):717–9. doi: 10.3201/eid2304.161886 (PMC5367432; doi:10.3201/eid2304.161886)
Supplement: Technical Appendix 1 — Materials and methods for clinical sample collection, virus isolation, and laboratory confirmation for the analysis of novel reassortant highly pathogenic avian influenza virus subtype H5N8 implicated in outbreaks among waterfowl at 2 zoos in India. Summary of avian influenza H5N8–positive samples. Maximum-likelihood phylogenetic trees for all 8 genes. Comparison of nucleotide sequence homology of H5N8 highly pathogenic avian influenza virus isolates in India compared with other available sequences in the database. Median-joining network analysis based on the hemagglutinin gene. [file 16-1886-Techapp-s1.pdf]

Article DOI: http://dx.doi.org/10.3201/eid2304.161886

# Novel Reassortant Highly Pathogenic Avian Influenza (H5N8) Virus in Zoos, India

## Technical Appendix 1

## Materials and Methods

### Outbreaks, Clinical Samples, Virus Isolation, and Confirmation

Mortality was observed in water fowls (ducks, goose, pelican and painted storks) at National Zoological Park, Delhi (28.6020° N, 77.2478° E) on 17th October, 2016 and in painted storks at Gandhi Zoological Park, Gwalior, Madhya Pradesh (26.2183° N, 78.1828° E) on 20th October, 2016.

A total of 83 samples from both the zoological parks were received during October 2016. Tissue samples from each dead bird were pooled and homogenized to make 10% suspension in PBS (pH 7.2). Tracheal and cloacal swabs, environmental and fecal samples were processed for virus isolation. Virus isolation was carried in 9–11-days old specific pathogen free embryonated chickens eggs (*1*).

Viral RNA was extracted from the clinical samples using QIAamp Viral RNA Mini Kit (Qiagen, Germany) as per the manufacturer’s instructions. Type A and HA subtype identification of influenza viruses were carried out using one step RT-PCR and real time RT-PCR (*2*,*3*). The NA subtyping was carried out by RT-PCR (*4*). Avian influenza H5N8 virus positivity from the two zoological parks is given below (Technical Appendix 1 Table 1).

### Genome sequencing

For molecular characterization of the H5N8 viruses, viral RNA was extracted from infected allantoic fluid using QIAamp Viral RNA Mini Kit (Qiagen, Germany) as per the manufacturer’s instructions. All the 8 genes of the viruses were amplified by RT-PCR using Platinum *Taq* High Fidelity (Invitrogen, USA) as described previously (*5*). The PCR amplified products were purified using QIAquick gel extraction kit (Qiagen, Germany). Sequencing of the

gel purified products were carried out using BigDye cycle sequencing kit, version 3.1 (Applied Biosystems, USA) in an ABI 3130 Genetic analyzer (Applied Biosystems, USA).

### Genetic Analysis

To know the evolutionary relationships of the H5N8 viruses isolated from waterfowls during October 2016 in India, homologous sequences were obtained by BLAST search at the GISAID (http://www.gisaid.org) and GenBank (http://ncbi.nlm.nih.gov) along with other reference sequences. Coding regions were aligned using BioEdit, ver. 7.2.5 (http://www.mbio.ncsu.edu/bioedit/bioedit.html). Tree reconstruction was carried out using MEGA 6 software (http://www.megasoftware.net). Maximum likelihood trees were reconstructed using GTR model of nucleotide substitution with γ-distributed rate variation among sites with 4 rate categories (4 discrete categories of γ) available in MEGA. Phylogenetic trees were statistically evaluated by bootstrap method using 1000 resampling datasets. A median-joining phylogenetic network was constructed by using NETWORK, version 5 (http://www.fluxus-engineering.com/sharenet.htm).

### Intravenous pathogenicity index (IVPI)

Intravenous pathogenicity index (IVPI) of two H5N8 viruses was carried out following the method described earlier (*1*). The experiment was carried out in the BSL-3 containment animal wing of ICAR-NIHSAD, Bhopal as per the guidelines of CPCSEA, Government of India with approval from Institutional Animal Ethics Committee (IAEC approval No. 61/IAEC/HSADL/12). Briefly, eight 4-weeks old SPF chickens (white leghorn) were inoculated intravenously with 1:10 dilution of infected allantoic fluid. Birds were observed daily for any clinical signs/mortalities, and scored accordingly (0, healthy; 1, sick; 2, severely sick and 3, dead).

### References


1. World Organization for Animal Health. Avian influenza (infection with avian influenza viruses). Chapter 2.3.4. In: OIE terrestrial manual. Paris: World Organization for Animal Health; 2015.

2. Nagarajan S, Tosh C, Murugkar HV, Venkatesh G, Katare M, Jain R, et al. Isolation and molecular characterization of a H5N1 virus isolated from a jungle crow (*Corvus macrohynchos*) in India. Virus Genes. 2010;41:30–6. PubMed http://dx.doi.org/10.1007/s11262-010-0477-4

3. Nagarajan S, Murugkar HV, Tosh C, Behera P, Khandia R, Jain R, et al. Comparison of a nucleoprotein gene based RT-PCR with real time RT-PCR for diagnosis of avian influenza in clinical specimens. Res Vet Sci. 2012;93:504–7. PubMed http://dx.doi.org/10.1016/j.rvsc.2011.06.005

4. Fereidouni SR, Starick E, Grund C, Globig A, Mettenleiter TC, Beer M, et al. Rapid molecular subtyping by reverse transcription polymerase chain reaction of the neuraminidase gene of avian influenza A viruses. Vet Microbiol. 2009;135:253–60. PubMed http://dx.doi.org/10.1016/j.vetmic.2008.09.077

5. Tosh C, Nagarajan S, Murugkar HV, Jain R, Behera P, Katare M, et al. Phylogenetic evidence of multiple introduction of H5N1 virus in Malda district of West Bengal, India in 2008. Vet Microbiol. 2011;148:132–9. PubMed http://dx.doi.org/10.1016/j.vetmic.2010.08.015

**Technical Appendix 1 Table 1.** Avian influenza H5N8 positive samples from zoological parks, India

| Name of the zoological park | Species | Type of samples | No. positive samples |
|---|---|---|---|
| National Zoological Park, Delhi | Painted stork (*Mycteria leucocephala)* | Carcass | 01 |
| | Domestic duck (*Anas platyrhynchos domesticus*) | | 01 |
| | Domestic goose (*Anser domesticus*) | | 01 |
| | House crow *(Corvus splendens)* | | 01 |
| | Great White Pelican (*Pelecanus onocrotalus)* | | 01 |
| | Domestic duck (*Anas platyrhynchos domesticus*) | Cloacal swab | 02 |
| | Domestic duck (*Anas platyrhynchos domesticus*) | Tracheal swab | 01 |
| | - | Environmental samples | 02 |
| Gandhi Zoological Park, | Painted stork (*Mycteria leucocephala)* | Carcass | 02 |
| Gwalior, | Painted stork (*Mycteria leucocephala)* | Tissue | 06 |
| Madhya Pradesh | Painted stork (*Mycteria leucocephala)* | Fecal sample | 02 |
| Total | | | **20** |

**Technical Appendix 1 Table 2.** Nucleotide sequence homology of H5N8 HPAI viruses isolates of India compared to available sequences in database

| Segment | Position | Virus with the highest nucleotide identity | Homology (%) | *EpiFlu/* GenBank accession number* |
|---|---|---|---|---|
| Isolate 1: A/duck/India/10CA01/2016(H5N8) | | | | |
| PB2 | 30–2309 | A/great_crested_grebe/Uvs-Nuur_Lake/341/2016(H5N8) | 99.4 | EPI773754 |
| PB1 | 25–2301 | A/common_tern_/Uvs-Nuur_Lake/26/2016(H5N8) | 99.4 | EPI836612 |
| | | A/Brown-headed Gull/Qinghai/ZTO6-MU/2016(H5N8) | | EPI774496 |
| PA | 25–2175 | A/Bar-headed Goose/Qinghai/BTY9-LU/2016(H5N8) | 99.5 | EPI774258 |
| HA | 29–1735 | A/great_crested_grebe/Uvs-Nuur_Lake/341/2016(H5N8) | 99.3 | EPI773757 |
| | | A/black-headed_gull/Tyva/41/2016(H5N8) | | EPI823756 |
| NP | 46–1542 | A/mallard/Republic of Georgia/13/2011(H6N2) | 98.6 | CY185580 |
| NA | 21–1433 | A/Brown-headed_Gull/Qinghai/ZTO6-MU/2016(H5N8) | 99.4 | EPI774500 |
| M | 26–1004 | A/great_crested_grebe/Uvs-Nuur_Lake/341/2016(H5N8) | 98.9 | EPI773760 |
| NS | 27–864 | A/black-headed_gull/Tyva/41/2016(H5N8) | 99.7 | EPI823760 |
| | | A/Bar-headed Goose/Qinghai/BTY9-LU/2016(H5N8) | | EPI774263 |
| Isolate 2: A/painted stork/India/10CA03/2016(H5N8) | | | | |
| PB2 | 30–2309 | A/great_crested_grebe/Uvs-Nuur_Lake/341/2016(H5N8) | 99.7 | EPI773754 |
| PB1 | 25–2301 | A/Brown-headed Gull/Qinghai/ZTO6-MU/2016(H5N8) | 99.5 | EPI774496 |
| PA | 25–2175 | A/northern shoveler/Georgia/1/2010(H2N3) | 98.2 | CY121988 |
| HA | 29–1735 | A/great_crested_grebe/Uvs-Nuur_Lake/341/2016(H5N8) | 99.3 | EPI773757 |
| | | A/black-headed_gull/Tyva/41/2016(H5N8) | | EPI823756 |
| | | A/Bar-headed_Goose/Qinghai/BTY17-LU/2016(H5N8) | | EPI774394 |
| | | A/mute_swan/Croatia/85/2016(H5N8) | | EPI864746 |
| NP | 46–1542 | A/mallard/Republic of Georgia/3/2010(H7N3) | 98.9 | CY185436 |
| NA | 21–1433 | A/Brown-headed_Gull/Qinghai/ZTO6-MU/2016(H5N8) | 99.8 | EPI774500 |
| M | 26–1004 | A/great_crested_grebe/Uvs-Nuur_Lake/341/2016(H5N8) | 99.0 | EPI773760 |
| NS | 27–864 | A/black-headed_gull/Tyva/41/2016(H5N8) | 99.5 | EPI823760 |
| | | A/Bar-headed Goose/Qinghai/BTY9-LU/2016(H5N8) | | EPI774263 |

*****EpiFlu Database of Global Initiative on Sharing All Influenza Data (GISAID).

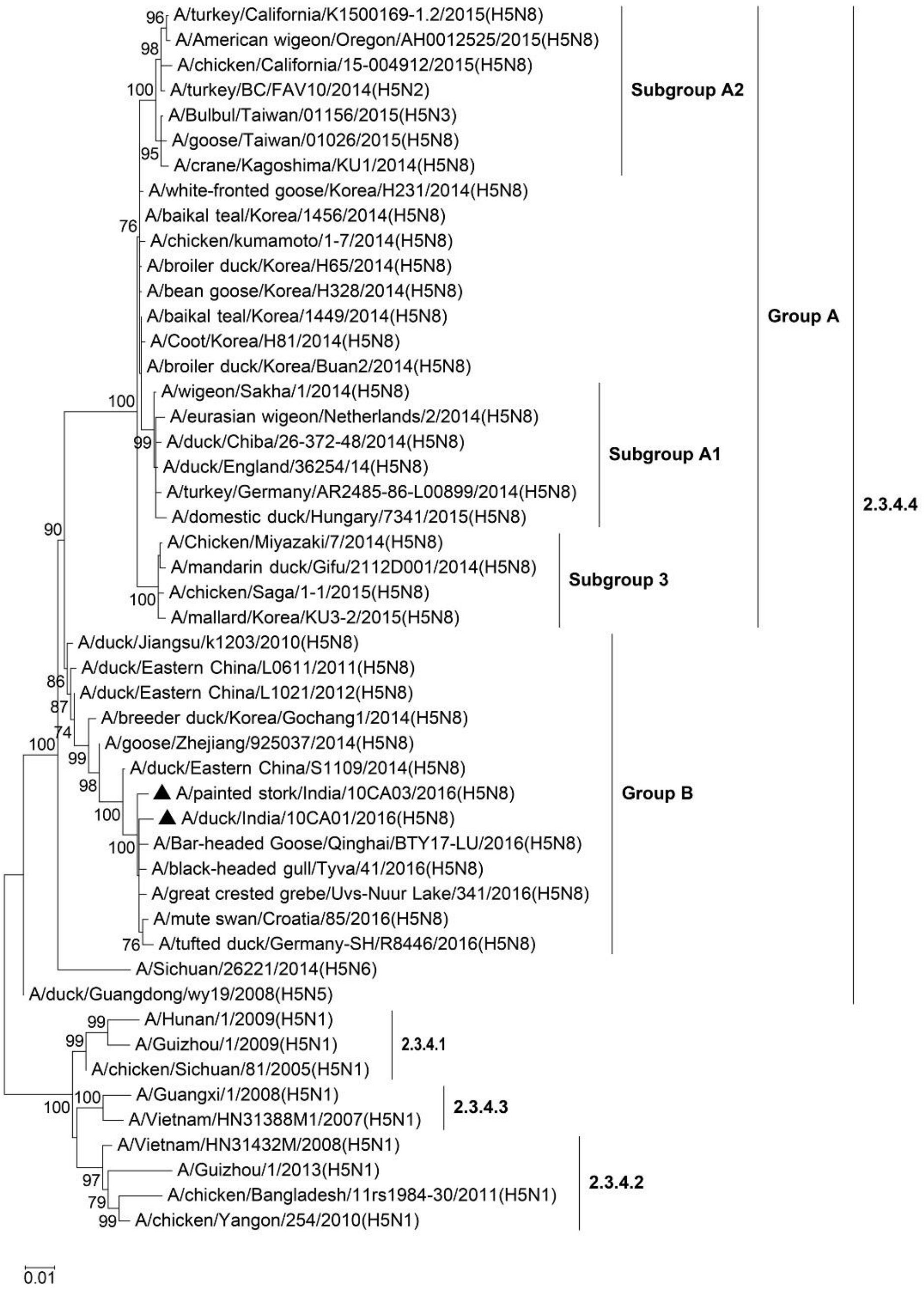


**Technical Appendix 1 Figure 1.** Maximum-likelihood phylogenetic tree of HA gene (nucleotide positions 29–1735) of influenza A/H5 viruses. Clades and genetic groups are shown to the right. Bootstrap values (≥70%) are shown near the nodes. Viruses sequenced in this study are highlighted with solid triangles. Scale bar indicates nucleotide substitution per site.

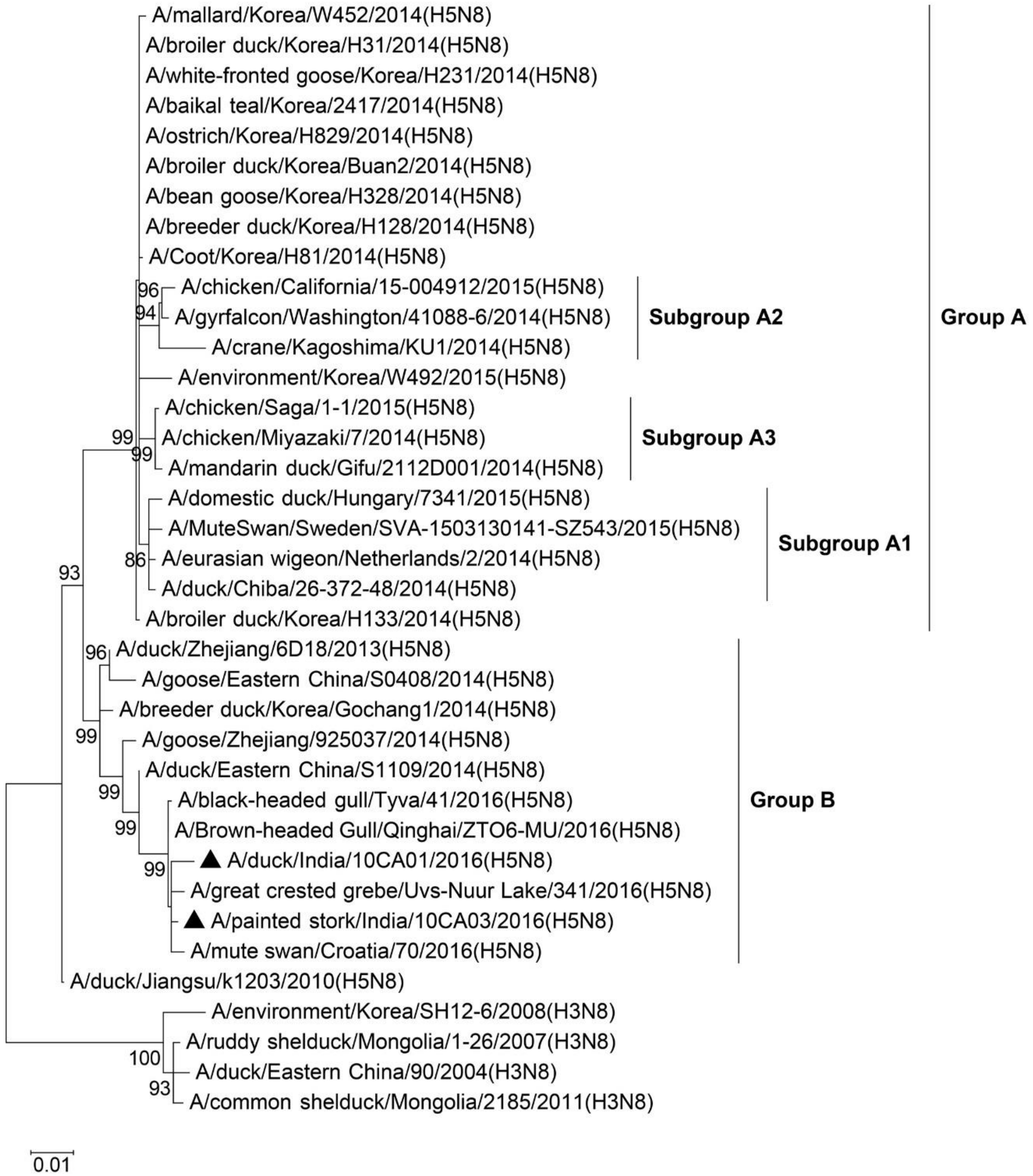


**Technical Appendix 1 Figure 2.** Maximum-likelihood phylogenetic tree of NA gene (nucleotide positions 21–1433) of influenza A/H5 viruses. Genetic groups are shown to the right. Bootstrap values (≥70%) are shown near the nodes. Viruses sequenced in this study are highlighted with solid triangles. Scale bar indicates nucleotide substitution per site.

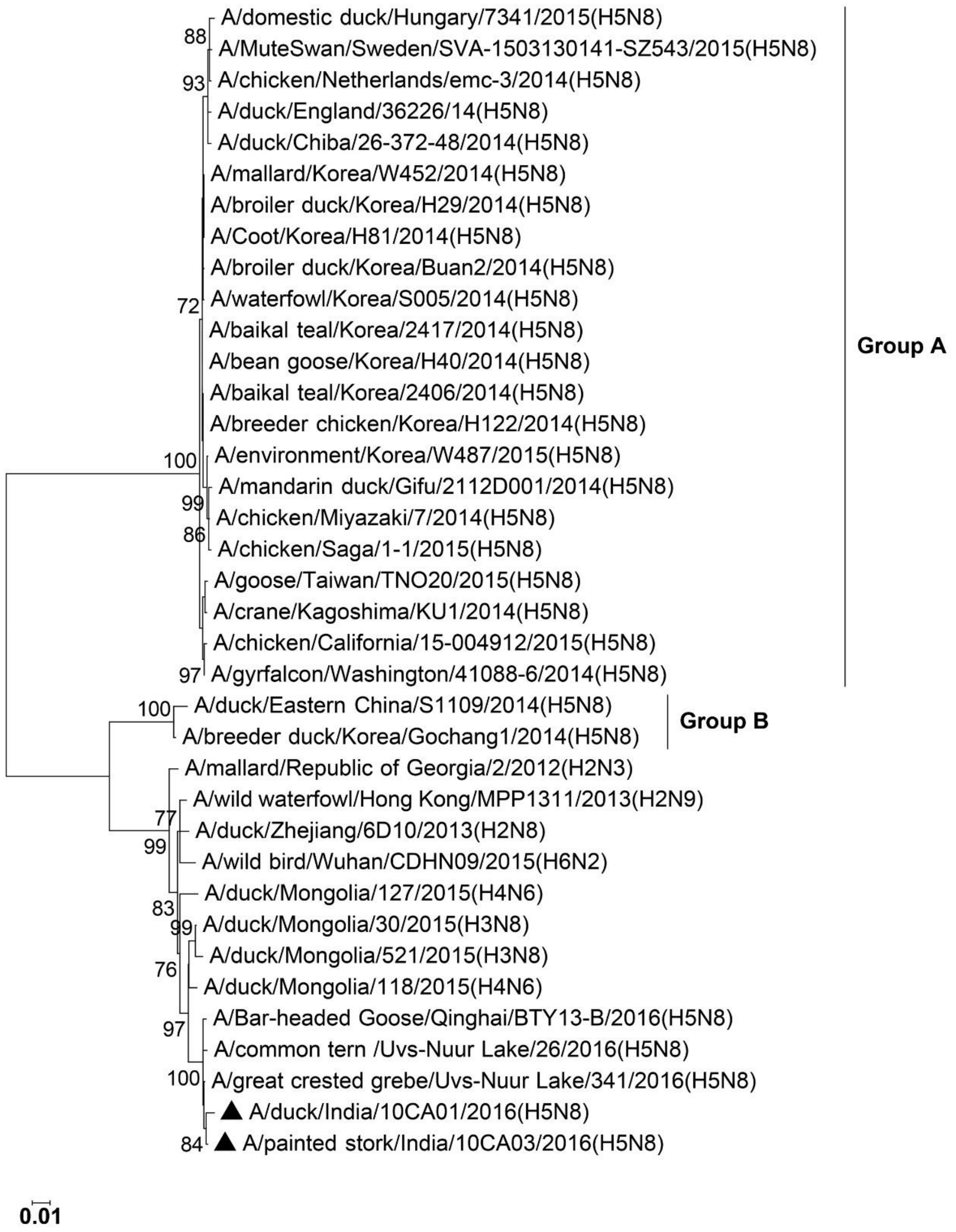


**Technical Appendix 1 Figure 3.** Maximum-likelihood phylogenetic tree of PB2 gene (nucleotide positions 30–2309) of influenza A/H5 viruses. Genetic groups are shown to the right. Bootstrap values (≥70%) are shown near the nodes. Viruses sequenced in this study are highlighted with solid triangles. Scale bar indicates nucleotide substitution per site.

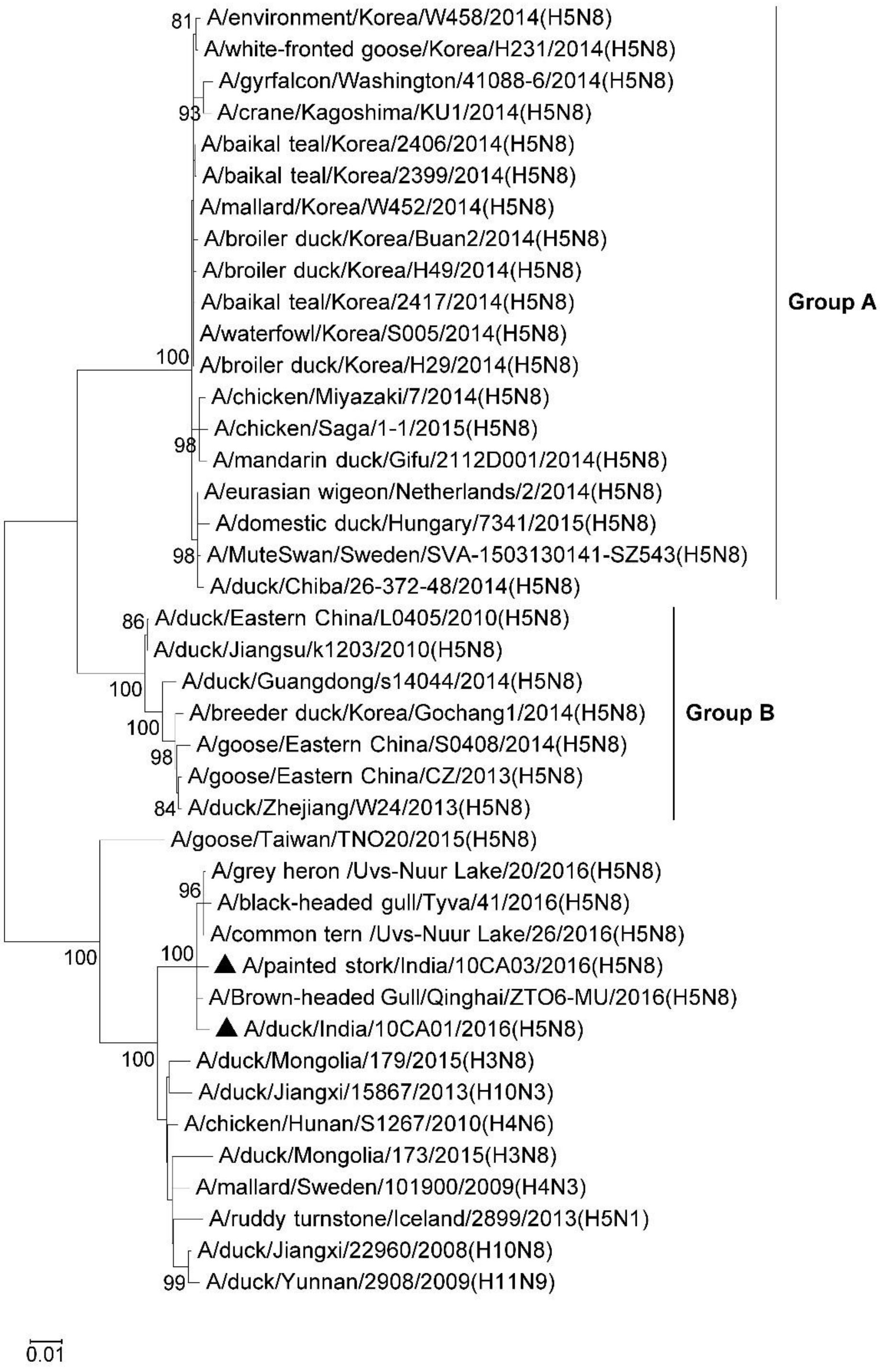


**Technical Appendix 1 Figure 4.** Maximum-likelihood phylogenetic tree of PB1 gene (nucleotide positions 25–2301) of influenza A/H5 viruses. Genetic groups are shown to the right. Bootstrap values (≥70%) are shown near the nodes. Viruses sequenced in this study are highlighted with solid triangles. Scale bar indicates nucleotide substitution per site.

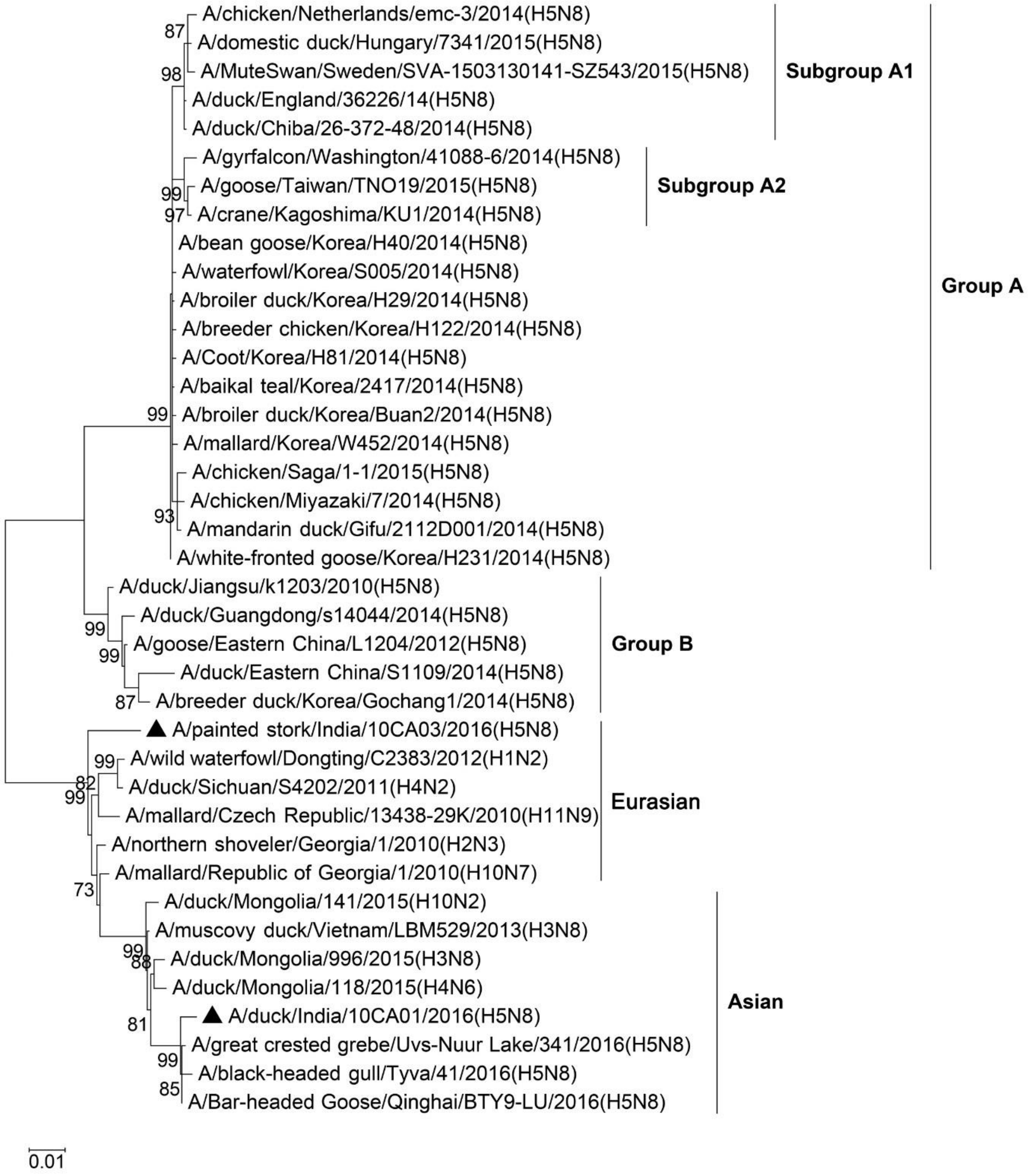


**Technical Appendix 1 Figure 5.** Maximum-likelihood phylogenetic tree of PA gene (nucleotide positions 25–2175) of influenza A/H5 viruses. Genetic groups are shown to the right. Bootstrap values (≥70%) are shown near the nodes. Viruses sequenced in this study are highlighted with solid triangles. Scale bar indicates nucleotide substitution per site.

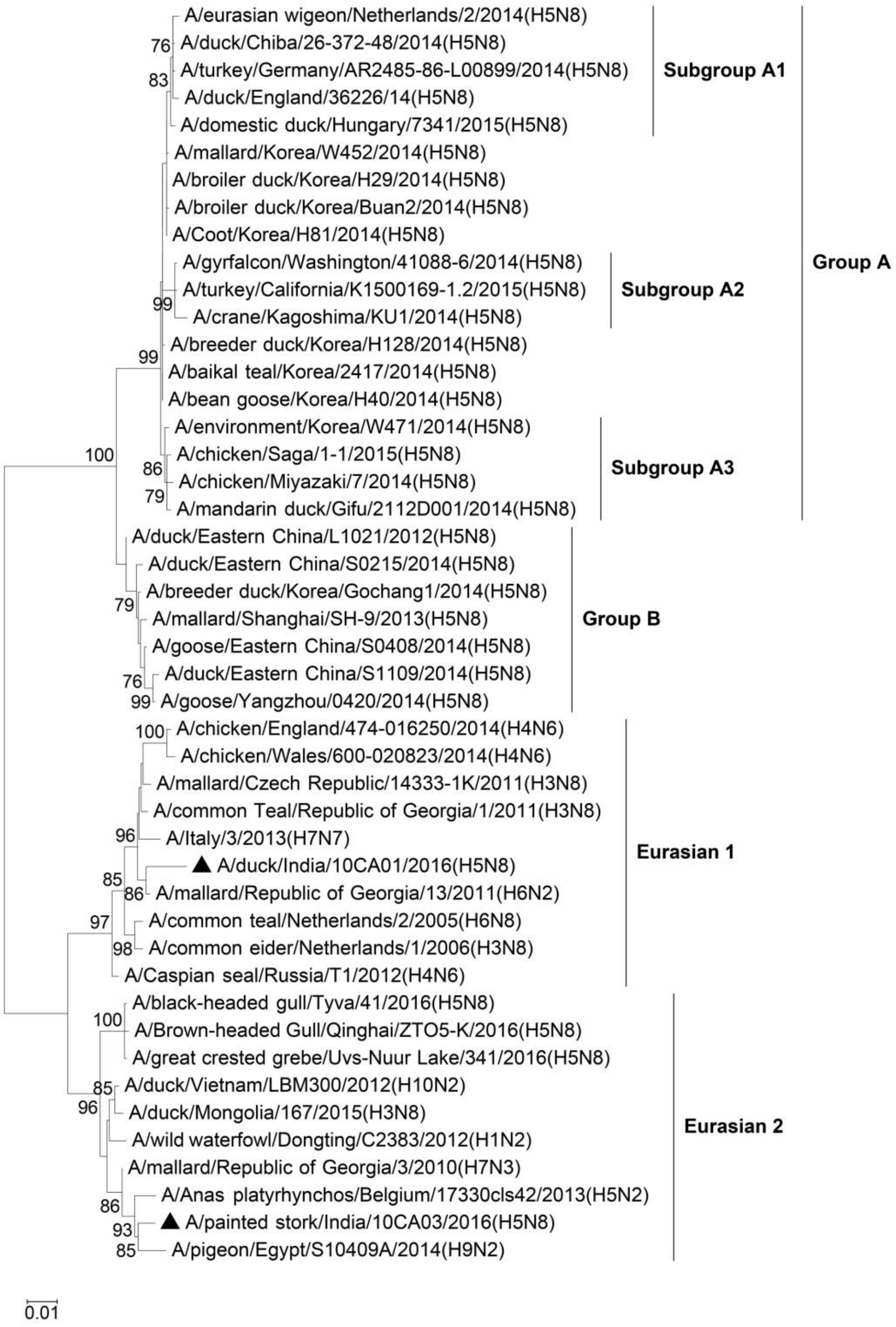


**Technical Appendix 1 Figure 6.** Maximum-likelihood phylogenetic tree of NP gene (nucleotide positions 46–1542) of influenza A/H5 viruses. Genetic groups are shown to the right. Bootstrap values (≥70%) are shown near the nodes. Viruses sequenced in this study are highlighted with solid triangles. Scale bar indicates nucleotide substitution per site.

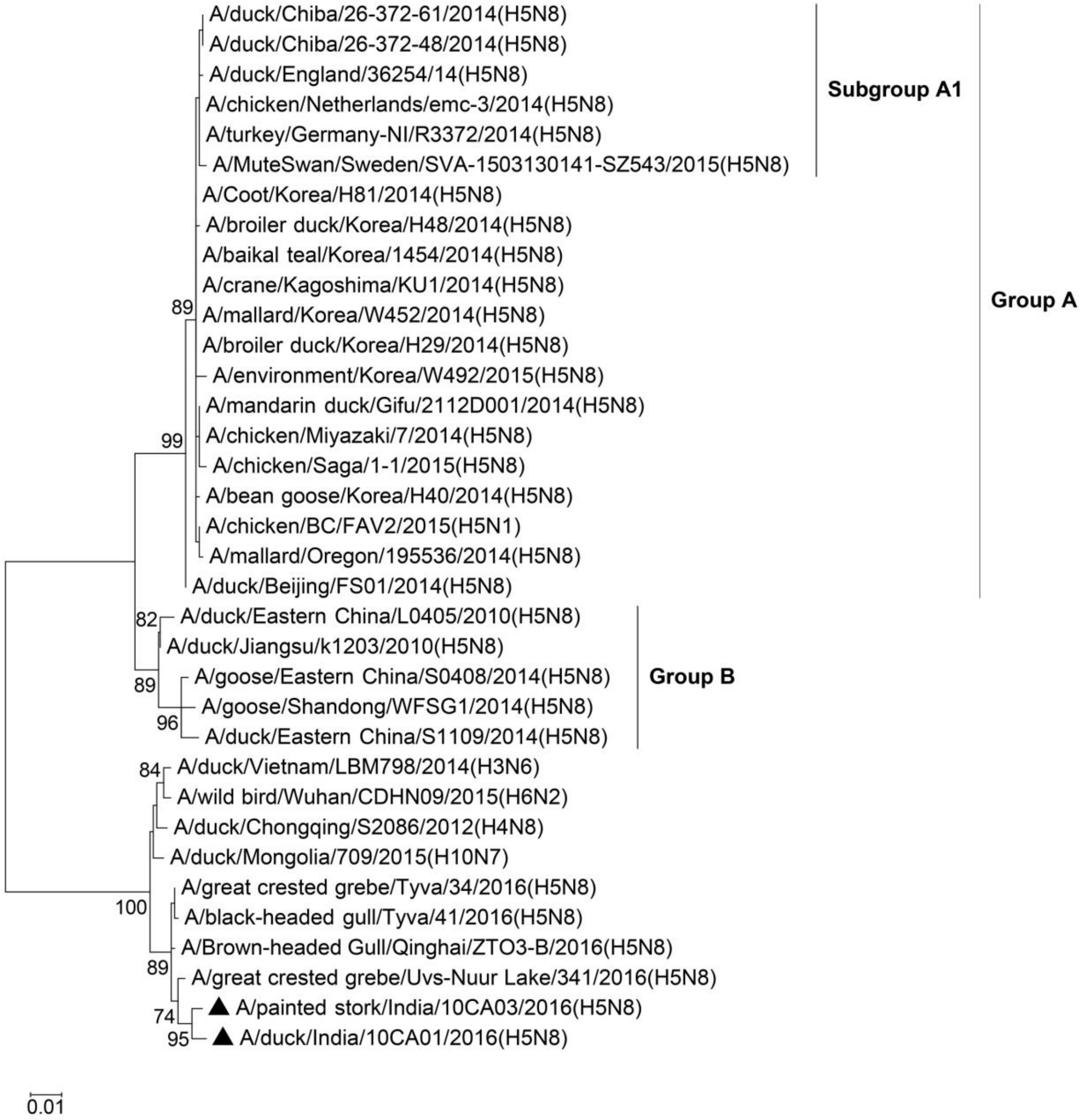


**Technical Appendix 1 Figure 7.** Maximum-likelihood phylogenetic tree of M gene (nucleotide positions26–1004) of influenza A/H5 viruses. Genetic groups are shown to the right. Bootstrap values (≥70%) are shown near the nodes. Viruses sequenced in this study are highlighted with solid triangles. Scale bar indicates nucleotide substitution per site.

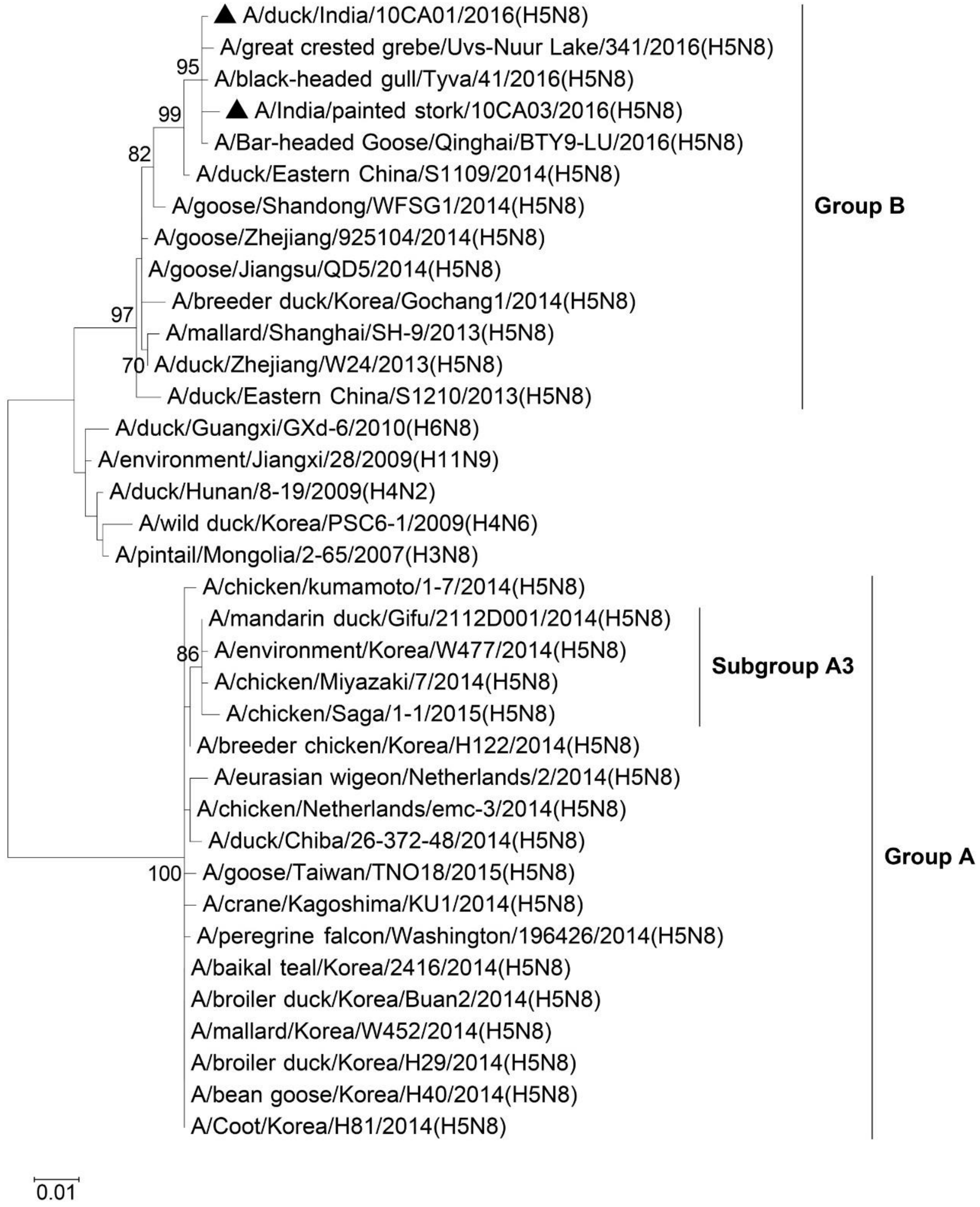


**Technical Appendix 1 Figure 8.** Maximum-likelihood phylogenetic tree of NS gene (nucleotide positions 27–864) of influenza A/H5 viruses. Genetic groups are shown to the right. Bootstrap values (≥70%) are shown near the nodes. Viruses sequenced in this study are highlighted with solid triangles. Scale bar indicates nucleotide substitution per site.

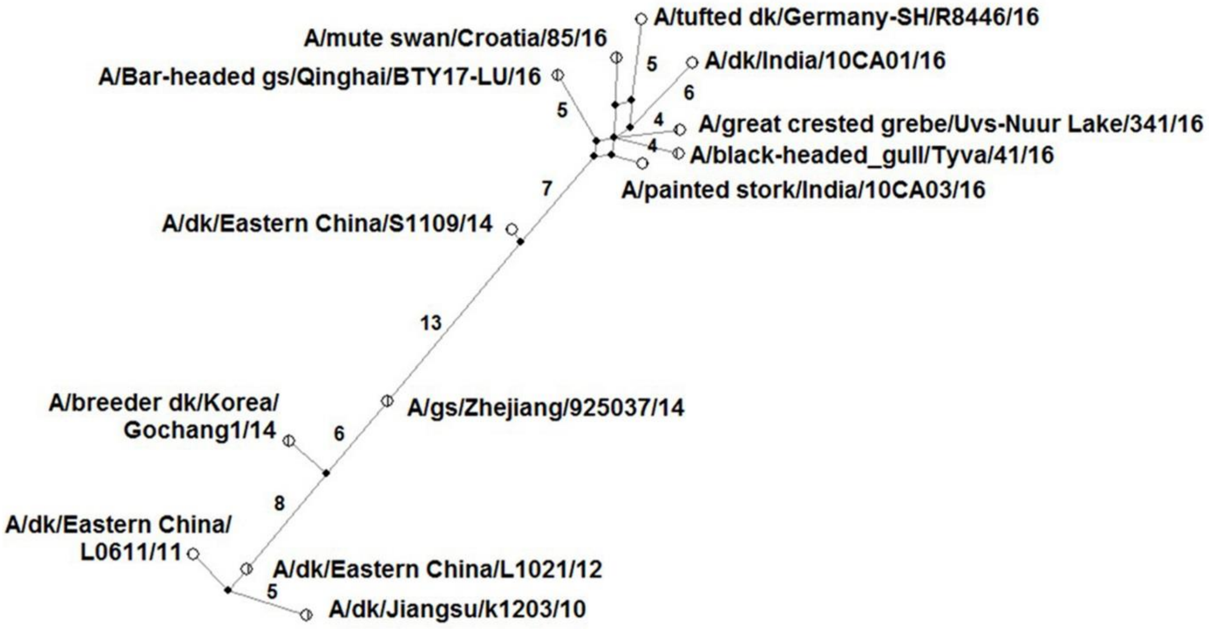


**Technical Appendix 1 Figure 9**. Median-joining phylogenetic network based on HA gene of H5N8 HPAI viruses of clade 2.3.4.4-B (as per HA gene phylogeny). Branch lengths are proportional to the number of mutations. Numbers along the branch represent the number of nucleotide substitutions distinguishing different nodes. Branches without number indicate 1–3 mutations. Dk, duck; Gs, goose.
